# Synthetic double-stranded RNA in antiviral immunity and vaccine adjuvant: insights into poly(I:C) and poly(A:U)

**DOI:** 10.3389/fimmu.2026.1839325

**Published:** 2026-07-07

**Authors:** Mohammad Enamul Hoque Kayesh, Michinori Kohara, Kyoko Tsukiyama-Kohara

**Affiliations:** 1 Department of Microbiology and Public Health, Faculty of Animal Science and Veterinary Medicine, Patuakhali Science and Technology University, Barishal, Bangladesh; 2 Transboundary Animal Diseases Center, Joint Faculty of Veterinary Medicine, Kagoshima University, Kagoshima, Japan; 3 Department of Microbiology and Cell Biology, Tokyo Metropolitan Institute of Medical Science, Tokyo, Japan

**Keywords:** antiviral immunity, innate immune signaling, poly(A:U), poly(I:C), synthetic dsRNA, vaccine adjuvant

## Abstract

Synthetic double-stranded RNA (dsRNA) analogues function as viral mimetics that activate innate immune signaling pathways critical for antiviral defense and the induction of adaptive immunity. Among dsRNA analogues, polyinosinic:polycytidylic acid [poly(I:C)] and polyadenylic:polyuridylic acid [poly(A:U)] have been extensively studied for their immunostimulatory properties and potential as vaccine adjuvants. This review examines how host pattern-recognition receptors sense synthetic dsRNA and explains the rationale for using poly(I:C) and poly(A:U) as representative dsRNA analogues with distinct structural and signaling properties. We compare their antiviral and adjuvant activities, emphasizing differences and commonalities in receptor engagement, downstream signaling, immunogenicity, and safety. Finally, we address emerging applications, translational challenges, and future directions for the rational design and clinical development of dsRNA-based immunomodulators in antiviral immunity and vaccine strategies.

## Graphical Abstract

**Figure:**
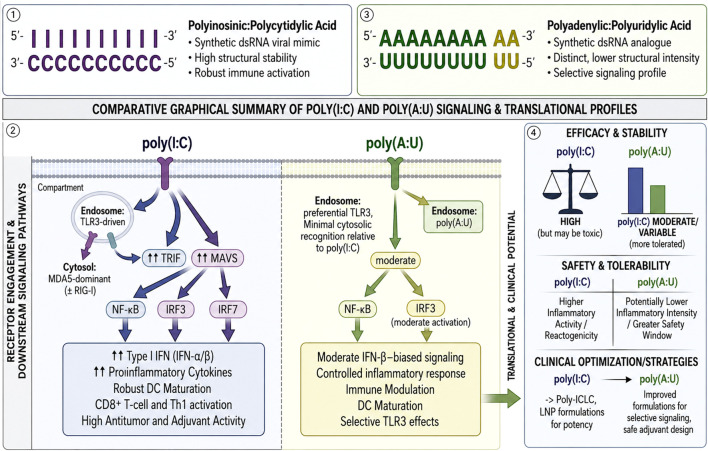

## 1 Introduction

Synthetic double-stranded RNAs (dsRNAs) exhibit potent immunostimulatory properties that can be exploited to induce antiviral immune responses and to enhance vaccine efficacy through rational adjuvant design (1–6). By mimicking viral RNA, synthetic dsRNAs are sensed by host pattern-recognition receptors (PRRs), a feature that underpins their effectiveness as antiviral agents and vaccine adjuvants (5, 7–9).

Among dsRNA analogues, polyinosinic:polycytidylic acid [poly(I:C)] and polyadenylic:polyuridylic acid [poly(A:U)] are widely studied synthetic analogues of viral dsRNA that act as pathogen-associated molecular patterns (PAMPs) to activate innate immune signaling pathways (5). Poly(I:C) is a well-established agonist of PRRs and a robust inducer of type I interferon (IFN) and pro-inflammatory responses, whereas poly(A:U) also activates innate immunity but generally elicits weaker IFN and cytokine responses across most systems (2, 5, 10, 11).

Recognition of viral and synthetic dsRNAs by endosomal TLR3 and cytosolic RNA sensors, including retinoic acid–inducible gene I (RIG-I) and melanoma differentiation-associated gene 5 (MDA5), enables poly(I:C) and poly(A:U) to induce type I IFN responses and downstream antiviral signaling pathways (10, 12–16). These pathways provide a broad antiviral state through the induction of IFN-stimulated genes (ISGs), including protein kinase R (PKR), 2′–5′-oligoadenylate synthetase (OAS), and RNase L, thereby suppressing viral replication (17, 18). Beyond antiviral activity, dsRNA-driven signaling modulates dendritic cell (DC) maturation, antigen presentation, and T-cell polarization, thereby shaping the magnitude and quality of subsequent adaptive immune responses and underscoring the central role of synthetic dsRNA at the interface of innate and adaptive immunity (10, 13).

Poly(I:C) has been widely reported as a vaccine adjuvant capable of enhancing both humoral and cellular immune responses when used alone or in combination with other adjuvants across multiple vaccine platforms, including malaria, influenza, and SARS-CoV-2 vaccines (19–23). Similarly, poly(A:U) has also been shown to stimulate both cellular and humoral immunity (24, 25).

A clear understanding of the shared and distinct innate immune mechanisms activated by synthetic dsRNAs is essential for optimizing their use in antiviral immunity and rational vaccine adjuvant design. Accordingly, a systematic comparison of poly(I:C) and poly(A:U) is particularly important, as differences in their structural and signaling properties can markedly influence efficacy, safety, and translational potential. This review compares the mechanisms of action and immunological effects of poly(I:C) and poly(A:U) as antiviral agents and vaccine adjuvants, with a focus on receptor engagement and length-dependent recognition, and how these features shape immune cell activation, cytokine induction, and antiviral signaling pathways. We further discuss emerging applications and future directions for dsRNA-based immunomodulators in antiviral immunity, vaccine development, and cancer immunotherapy, with emphasis on their rational translational use.

## 2 Receptor engagement and length-dependent recognition of poly(I:C) and poly(A:U)

Poly(I:C) is a synthetic dsRNA generated by annealing the homopolyribonucleotides polyinosinic acid (poly-I) and polycytidylic acid (poly-C), yielding a stable double-helical structure (26). In contrast, Poly(A:U) consists of complementary polyadenylic and polyuridylic acid strands that form a synthetic polyribonucleotide complex with lower thermodynamic stability (27).

Scavenger receptors mediate the cellular uptake of extracellular dsRNA by facilitating the endocytosis of poly(I:C), promoting its delivery to endosomal TLR3 and enhancing downstream nuclear factor-kappa-light-chain-enhancer of activated B cells (NF-κB), mitogen-activated protein kinase (MAPK), and IFN regulatory factor 3 (IRF3) signaling (28). Both poly(I:C) and poly(A:U) are recognized by the cytosolic RNA sensor MDA5; however, poly(I:C) exhibits higher binding affinity and signaling potency, consistent with its stronger induction of antiviral responses (14, 15). In addition to MDA5, poly(I:C) can also activate RIG-I, leading to robust induction of type I IFNs and pro-inflammatory cytokines (29, 30). By comparison, the reduced stability of poly(A:U) likely limits its ability to efficiently engage these innate immune sensors, resulting in weaker downstream signaling.

The immunostimulatory activity of poly(I:C), including its ability to induce type I IFNs and confer antiviral resistance, is strongly dependent on molecular length. Short (~1–1.5 kb) and long (>5 kb) poly(I:C) species preferentially engage distinct PRRs, producing cell-type–specific signaling outcomes (31). Long poly(I:C) induces robust cytokine production and antiviral responses in fibroblasts but elicits comparatively weaker responses in myeloid cells. Conversely, short poly(I:C) induces higher levels of tumor necrosis factor alpha (TNF-α), IL-8, and IFN-β and promotes stronger antiviral activity in myeloid cells, while exhibiting limited efficacy in fibroblasts (31).

Early studies showed that higher-molecular-weight poly(I:C) elicits stronger IFN responses, although the magnitude of IFN induction and antiviral resistance varies across cell types (32). Consistent with this, high-molecular-mass poly(I:C) activates TLR3 more efficiently than low-molecular-mass poly(I:C) (33). During viral infection, long dsRNA intermediates generated during replication serve as principal triggers of IFN induction, underscoring the physiological relevance of dsRNA length in innate immune activation (34). Consistent with these observations, both the immunological efficacy and safety profile of poly(I:C) are highly length dependent, with polymer size critically influencing therapeutic benefit and the risk of excessive immune activation (35).

Ligand length directly determines receptor recognition and signaling efficiency. Endosomal TLR3 preferentially detects long dsRNA, as its ectodomains require dsRNA molecules of at least ~40–50 base pairs to dimerize and initiate downstream signaling (36–38). Longer dsRNA ligands further promote cooperative clustering of TLR3 dimers along the RNA helix, facilitating efficient assembly of downstream signaling complexes and amplifying innate immune responses (39). However, uncontrolled length extension during strand annealing complicates mechanistic analyses and limits precise modulation of immune signaling. This challenge was addressed by Nakano et al., who developed an asymmetric strand assembly strategy to generate length-defined poly(I:C) with preserved receptor responsiveness (35).

Cytosolic RNA sensors likewise exhibit distinct length preferences. RIG-I preferentially recognizes RNA species bearing a 5′-triphosphate moiety and can also respond to relatively short dsRNA fragments (approximately 300–1000 bp) lacking this modification (15, 40, 41). Short poly(I:C) (<500 bp) efficiently activates RIG-I–mediated antiviral signaling, whereas long poly(I:C) (>500 bp), despite binding to RIG-I, fails to trigger productive signaling (42, 43). Mechanistically, RIG-I binds long dsRNA with slow kinetics and forms stable, nonproductive complexes, while short dsRNA promotes ATP-dependent dissociation and receptor oligomerization required for signaling activation (44).

In contrast, MDA5 is selectively activated by long dsRNA and responds inefficiently to short ligands. Binding of long dsRNA, including high-molecular-weight poly(I:C), induces cooperative assembly of MDA5 filaments along the RNA duplex, a process essential for robust downstream signaling and antiviral innate immune activation (30, 45). This differential length-dependent recognition by RIG-I and MDA5 highlights the critical role of poly(I:C) molecular size in shaping innate immune signaling pathways.

Functionally, poly(I:C) length is a key determinant of immunostimulatory efficacy. Progressive shortening of the polymer markedly reduces IFN induction, antiviral protection, and adjuvant activity, with vaccine adjuvant effects being particularly sensitive to dsRNA length. Importantly, reductions in polymer size do not proportionally attenuate adverse effects, such as inhibition of hepatic drug-metabolizing enzymes, highlighting an intrinsic trade-off between immune activation and toxicity (46).

Collectively, these findings demonstrate that both the structural composition and molecular length of synthetic dsRNAs critically shape receptor engagement, downstream signaling, and immunostimulatory potency. In this context, the greater stability and length-dependent recognition of poly(I:C) contribute to its stronger activation of innate immune pathways compared with poly(A:U). Notably, the immunostimulatory activity of poly(I:C) in intranasal influenza vaccination is highly dependent on dsRNA length, with optimized chain composition enhancing efficacy while reducing toxicity. A rationally engineered short-chain poly(I:C) variant (uPIC100-400) selectively engages TLR3, induces robust mucosal IgA responses, and limits viral replication, underscoring the importance of length-dependent TLR3 recognition in dsRNA-adjuvanted vaccines (47).

## 3 Poly(I:C) as antiviral and vaccine adjuvant

**Figure 1:**
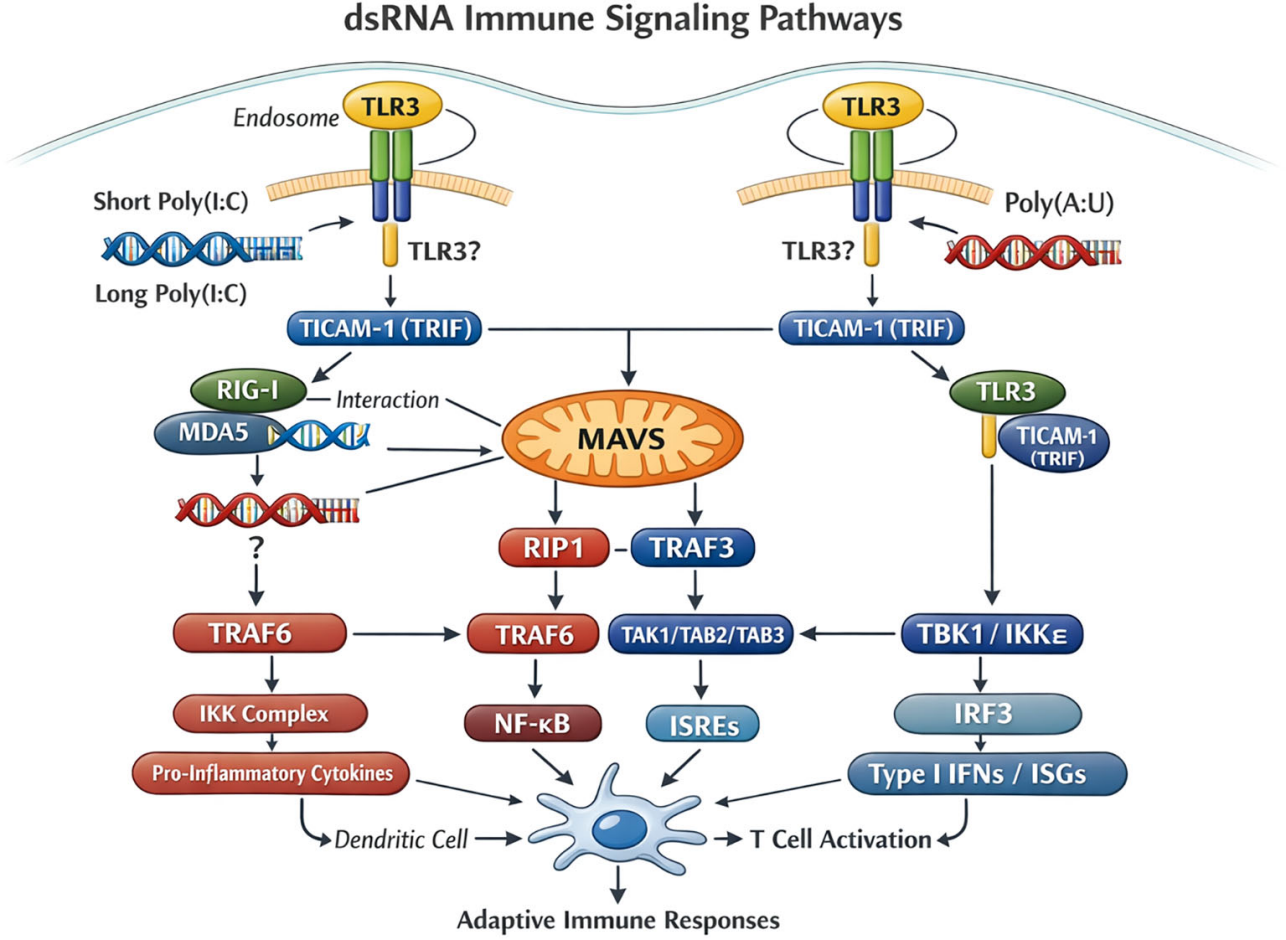
Immunostimulatory signaling of poly(I:C) and poly(A:U) via dsRNA sensors. Poly(I:C) activates endosomal TLR3, recruiting the adaptor TICAM-1 (TRIF), or cytoplasmic MDA5 and RIG-I, which signal through MAVS. Poly(A:U) primarily engages TLR3. Both pathways induce pro-inflammatory cytokine and type I interferon (IFN) responses in innate immune cells, leading to dendritic cell–mediated T-cell activation and adaptive immune responses. Question marks indicate receptor engagements that remain to be fully defined. TLR3, Toll-like receptor 3; MDA5, melanoma differentiation-associated protein 5; RIG-I, retinoic acid–inducible gene I; TICAM-1, TIR-domain-containing adapter molecule 1; TRIF, TIR-domain-containing adapter-inducing interferon-β; MAVS, mitochondrial antiviral signaling protein; TRAF3, TNF receptor-associated factor 3; TRAF6, TNF receptor-associated factor 6; RIP1, receptor-interacting protein 1; TBK1, TANK-binding kinase 1; TAB2, TAK1-binding protein 2; TAB3, TAK1-binding protein 3; IKKϵ, IκB kinase epsilon; IRF3, interferon regulatory factor 3; NF-κB, nuclear factor kappa-light-chain-enhancer of activated B cells; ISRE, interferon-stimulated response element; ISGs, interferon-stimulated genes.

Both poly(I:C) and poly(A:U) exert their antiviral and adjuvant activities primarily through activation of innate immune receptors (**Figure 1**; **Table 1**), with poly(I:C) generally inducing stronger type I IFN responses and DC maturation. The antiviral activity of poly(I:C) is conserved across vertebrates and is mediated through TLR3- and MDA5-dependent IFN signaling pathways. This evolutionary conservation has been demonstrated in teleost fish models, where poly(I:C) enhances antiviral defenses (48), as well as in mammalian airway epithelial cells, where it compensates for impaired IFN responses and increases resistance to rhinovirus infection (49). In influenza models, poly(I:C) has shown both prophylactic and therapeutic efficacy, reducing viral loads and improving survival following challenge (50).

**Table 1:** Comparative immunostimulatory profiles of poly(I:C) and poly(A:U).

Feature	Poly(I:C)	Poly(A:U)
Primary PRR	TLR3, MDA5	TLR3
Type I IFN induction	High (e.g., IFN-β ~700–1000 pg/mL within 4–8 h in epithelial cells)	Lower than poly(I:C) (relative activity often <50% of poly(I:C))
IRF3/NF-κB activation	Strong (multi-fold cytokine induction)	Moderate; primarily TLR3-dependent signaling only
ISG expression	Strong (robust induction of ISGs such as OAS, MxA with sustained expression up to 24 h)	Moderate (reduced downstream signaling due to lack of MDA5/RIG-I activation)
DC maturation	Strong (robust TLR3 and cytosolic receptor signaling with enhanced activation)	Moderate (TLR3-dependent only; reduced signaling breadth)
Systemic inflammation	High (e.g., IL-6 and proinflammatory cytokines increased 2–3 fold)	Low (weaker cytokine and cytotoxic response)

PRR, pattern recognition receptor; TLR3, Toll-like receptor 3; MDA5, melanoma differentiation-associated protein 5; RIG-I, retinoic acid–inducible gene I; IRF3, interferon regulatory factor 3; NF-κB, nuclear factor kappa-light-chain-enhancer of activated B cells; ISG, interferon-stimulated gene; OAS, 2′-5′-oligoadenylate synthetase; MxA, myxovirus resistance protein A; DC, dendritic cell.

Poly(I:C), a potent TLR3 agonist, elicits type I IFN responses and induces antiviral gene expression that inhibits viral replication across multiple cell types. In CHIKV-infected human bronchial epithelial cells, poly(I:C) suppresses viral replication and cytopathic effects through TLR3-mediated induction of IFN-β and downstream antiviral effectors, including IFN-α, Myxovirus resistance protein A (MxA), and OAS (51). Consistently, poly(I:C) treatment also induces IFN-β expression in human bronchial epithelial cells during influenza virus infection (52).

In inflammatory macrophages, poly(I:C) exerts antiviral effects through IFN-β; neutralization of IFN-β abolishes protection, whereas exogenous IFN-β alone is sufficient to confer resistance to HSV-1 replication (53). This IFN-β acts in an autocrine manner to activate downstream antiviral effectors, including OAS, thereby restricting HSV-1 replication (54). Consistent with this mechanism, TLR3-dependent poly(I:C) signaling leading to IFN-β expression has also been demonstrated in hepatocyte-derived cell models (55).

Poly(I:C) inhibits HIV replication in immature DCs, even when administered up to 60 h post-exposure, by inducing type I IFN and IL-12 production. This antiviral effect is mediated through IFN-α/β signaling and is associated with activation of low-molecular-mass APOBEC3G complexes, highlighting poly(I:C) as a potential strategy to limit early HIV dissemination (56). Poly(I:C) treatment of mesenchymal stem cells induces the upregulation of antiviral and immunomodulatory pathways, characterized by enhanced expression of cytokines including CCL2, CXCL10, CXCL11, CXCL8, and interleukin-6 (IL-6) (57).

A recent study demonstrated that a single intranasal administration of poly(I:C) confers strong protection against SARS-CoV-2 by rapidly priming lung innate immunity and inducing transient upregulation of antiviral genes. This early immune activation significantly reduces viral load, prevents cytokine storm development in the lung and brain, enhances macrophage and NK cell responses, and markedly improves survival in lethally infected K18-hACE2 mice (58). Similarly, poly(I:C)-induced immune priming protects Pacific oyster (*Crassostrea gigas*) from Ostreid herpesvirus (OsHV-1 μvar) infection by limiting viral replication and activating a conserved antiviral gene network consistent with a type I IFN-like innate immune response (59). Together, these studies support a model in which poly(I:C) suppresses viral replication through IFN-dependent induction of antiviral genes across diverse host systems.

**Figure 2:**
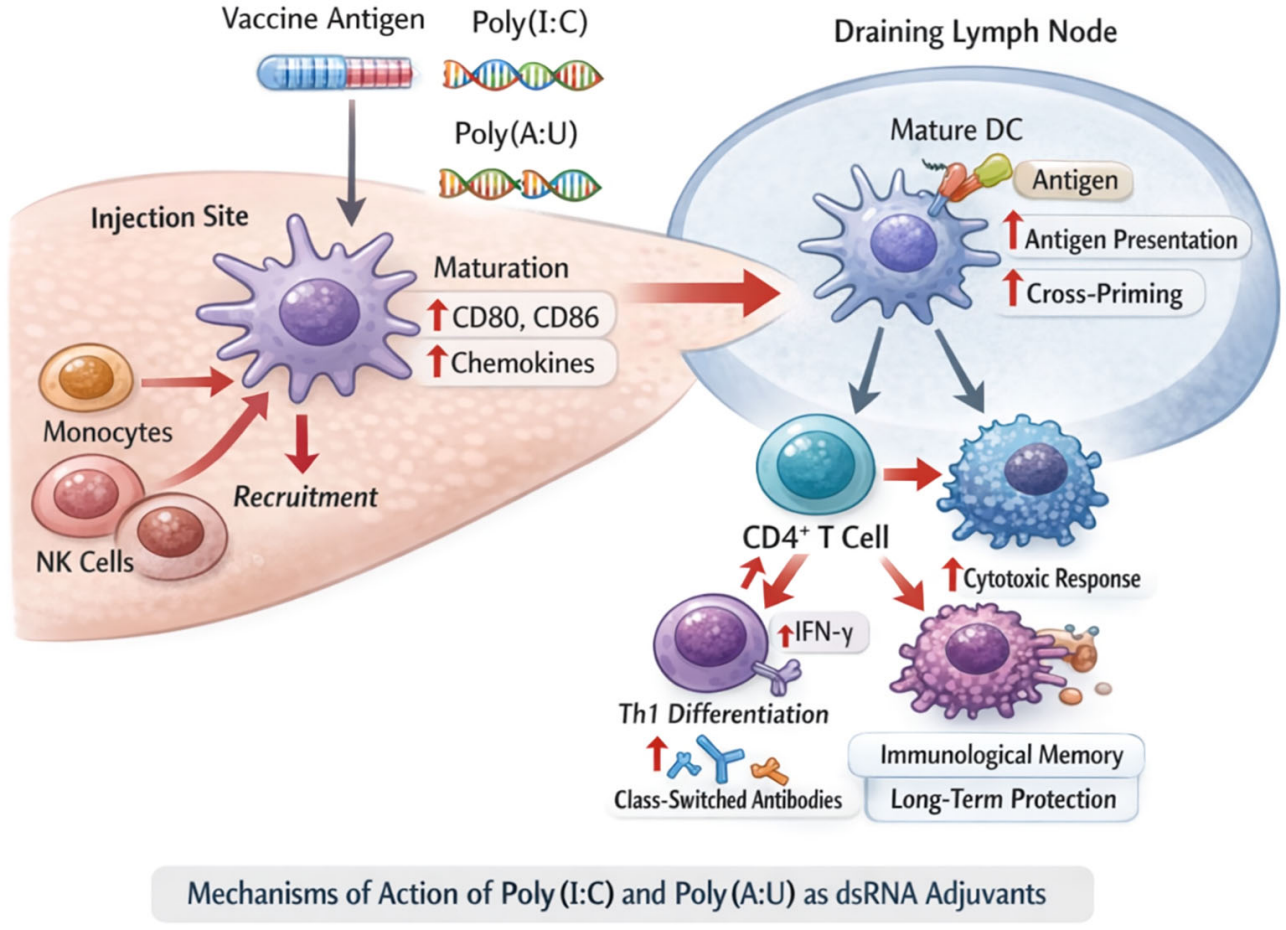
Mechanisms of action of poly(I:C) and poly(A:U) as dsRNA-based vaccine adjuvants. Upon co-administration with vaccine antigens, poly(I:C) and poly(A:U) enhance immune activation at the injection site by promoting dendritic cell (DC) maturation, upregulation of co-stimulatory molecules (CD80, CD86), and chemokine-driven recruitment of monocytes and natural killer (NK) cells. Activated DCs subsequently migrate to the draining lymph nodes (LN), where they improve antigen presentation and cross-priming. These processes drive Th1-polarized CD4^+^ T-cell differentiation and robust CD8^+^ cytotoxic T-lymphocyte (CTL) responses while also supporting class-switched antibody production. Poly(I:C) is particularly associated with strong cross-priming capacity and robust CTL induction, whereas poly(A:U), which primarily signals through TLR3, preferentially supports Th1-biased helper T-cell responses. Together, the coordinated induction of cellular and humoral immunity promotes the establishment of durable immunological memory. Differences in receptor engagement may further modulate the magnitude and breadth of these immune responses. Red arrows indicate activation, upregulation, or enhanced effects, as appropriate.

Poly(I:C) exerts its adjuvant activity primarily through activation of innate immune receptors (**Figure 2**). Poly(I:C) acts as a potent Th1-polarizing vaccine adjuvant by inducing a strong type I IFN–dependent response in the lymph node, leading to IFN-γ secretion and localized upregulation of the chemokine CXCL9 in the interfollicular region (60). This chemokine signature is associated with IgG2c antibody polarization and highlights a distinct innate signaling pathway through which poly(I:C) enhances adaptive immune responses compared with alum-based adjuvants (60).

Poly(I:C) is a particularly effective vaccine adjuvant for simian immunodeficiency virus (SIV), as priming with SIV Gag protein plus poly(I:C) induces significantly stronger Th1-biased CD4^+^ T cell responses than other tested TLR ligands (61). In a heterologous prime–boost regimen, poly(I:C) outperforms alternative adjuvants by enhancing the magnitude, breadth, and durability of SIV-specific cellular immunity and is associated with partial control of early SIVmac251 viral replication following challenge (61).

Its effects depend on TLR3 and MDA5 signaling, whereas its analogue poly IC_12_U requires only TLR3 (62). Co-stimulation with other innate immune agonists enhances its activity. For example, poly(I:C) combined with zymosan, a TLR2 ligand, synergistically activates DCs, increasing cytokine production, upregulating co-stimulatory molecules (CD86 and CD40), and improving protective immune responses (63). Similarly, poly(I:C) combined with monophosphoryl lipid A (MPL), a TLR4 ligand, augments antigen-specific antibody responses and memory T- and B-cell immunity while reducing inflammatory pathology following influenza infection (19).

Poly(I:C) has been evaluated in combination with other adjuvants, with evidence showing that co-administration with R848, a TLR7/8 agonist, enhances antigen-specific immune responses by promoting the activation and differentiation of B and T lymphocytes (64). In the context of foot-and-mouth disease virus (FMDV) vaccination, poly(I:C) combined with aluminum hydroxide–based formulations drives robust Th1-biased humoral and cellular immunity and compared with conventional adjuvant systems, significantly increases FMDV-specific antibody titers, CD8^+^ T-cell responses, and IFN-γ production, highlighting its superior capacity to elicit antiviral immune responses (65).

Poly(I:C) enhances DC–targeted protein vaccines, eliciting robust, multifunctional CD4^+^ Th1 responses with sustained cytokine production and proliferative capacity, particularly at mucosal sites (62, 66), as reported in HIV vaccine development.

Poly(I:C) is a potent mucosal adjuvant, especially for intranasal vaccination. It increases systemic and mucosal antibody responses to influenza vaccines, enhances pulmonary resident memory CD8^+^ T cells, and provides protective immunity following intranasal, but not intramuscular, delivery (67). Co-administration with chemokine CCL21 further amplifies antibody and T-cell responses while reducing pulmonary inflammation (68).

Intranasal poly(I:C) also promotes cross-protective immunity against variant H1N1 strains (4) and heterologous swine influenza virus (69). Intranasal vaccination with a pre-pandemic H5N1 vaccine adjuvanted with the TLR3 agonist poly(I:C_12_U) (Ampligen) induces robust mucosal IgA and systemic IgG responses and confers protection against both homologous and heterologous H5N1 challenge in mice, highlighting its potential as a safe and effective mucosal adjuvant for pandemic influenza vaccines (70). Timing of administration is also critical; as observed in mice, poly(I:C) given 24 hours post-vaccination enhanced protection, whereas simultaneous administration impaired immunity (71).

Extending the adjuvant efficacy of poly(I:C) across species, it significantly enhances the immunogenicity of inactivated H9N2 avian influenza vaccines in ducks by accelerating antibody responses, increasing antibody titers, and reducing viral shedding following challenge with antigenic variants (72). This enhanced protection is associated with elevated expression of antiviral and immune activation markers, including type I and II IFNs, IL-6, and MHC-II, demonstrating the capacity of poly(I:C) to boost both humoral and cellular immunity in avian hosts (72).

In murine models, intradermal vaccination with subunit viral antigens adjuvanted with poly(I:C) induces durable systemic and mucosal antibody responses and provides protection against genital herpes simplex virus type 2 (HSV-2) infection with minimal local reactogenicity (73). In contrast, the TLR3-restricted analogue poly(I:C_12_U) (Ampligen) elicits weaker IFN responses, fails to induce protective mucosal immunity, and does not confer protection, underscoring the importance of robust TLR3/MDA5 signaling in poly(I:C)-mediated adjuvant efficacy.

In the context of hepatitis B virus (HBV) vaccination, poly(I:C)/alum adjuvant priming enhances antigen-specific antibodies and induces Th1-biased CD4^+^ and CD8^+^ T-cell responses (74). As demonstrated in a non-human primate model of SARS-CoV-2 vaccination, sublingual poly(I:C)-adjuvanted recombinant receptor-binding domain (RBD) vaccines induce mucosal IgA and systemic IgG/IgA responses with minimal IgE production, supporting their safety for mucosal applications (20). Sublingual administration of poly(I:C) avoids upregulation of inflammatory genes in mice and macaques, unlike intranasal delivery, which transiently induces neuroinflammatory markers in the olfactory bulb and pons (75). Overall, poly(I:C) consistently enhances immune responses without triggering excessive inflammation, particularly at mucosal sites. Together with its ability to elicit robust antigen-specific mucosal and systemic antibody responses, these findings support the safety and effectiveness of poly(I:C) as a mucosal vaccine adjuvant.

In other vaccine platforms, poly(I:C) has also shown efficacy as an adjuvant. For example, poly(I:C) with circumsporozoite protein vaccines for *Plasmodium vivax* elicits high-titer IgG responses, promotes B-cell differentiation, and enhances plasma and memory B-cell generation, surpassing Alhydrogel in efficacy (21). In a zebrafish model, both free poly(I:C) and chitosan-stabilized poly(I:C) induce antiviral states by upregulating TLRs, IFNs, and IFN-stimulated genes (ISGs) (76).

While much of the evidence for poly(I:C) comes from preclinical studies, its immunostimulatory mechanisms—robust Th1 polarization, enhancement of systemic and mucosal antibodies, and synergistic potential with other adjuvants—provide a strong rationale for clinical translation, particularly in mucosal vaccines against influenza, SARS-CoV-2, and other pathogens. Overall, extensive *in vitro* and preclinical evidence demonstrates that poly(I:C) is a potent and versatile vaccine adjuvant that enhances innate activation and promotes robust systemic and mucosal humoral and cellular immunity across diverse vaccine platforms, with efficacy and safety strongly influenced by route, timing, and combination with complementary adjuvants.

## 4 Poly(A:U) as antiviral and vaccine adjuvant

Among synthetic dsRNA analogues, poly(A:U) has attracted attention for its antiviral activity and potential application as a vaccine adjuvant. Poly(A:U) exhibits strong antiviral activity against human immunodeficiency virus types 1 and 2 (HIV-1 and HIV-2), markedly delaying cytopathic effects and reducing viral protein production by up to 95%, likely through inhibition of viral entry, and shows synergistic effects with azidothymidine while exhibiting minimal toxicity (77). Subsequent studies demonstrated that both poly(A:U) and poly(I:C) inhibit HIV-1 and HIV-2 by blocking viral entry into CD4^+^ T lymphocytes (78). In contrast, poly(A:U) alone shows minimal antiviral activity against vesicular stomatitis virus, but this activity is potentiated 16- to 20-fold when combined with *N*^2^-methyl-9-hydroxyl-ellipticine or *N*^2^,*N*^6^-dimethyl-9-hydroxy-ellipticine (79).

Weekly intravenous administration of poly(A:U) at a dose of 100–150 mg for six weeks showed antiviral efficacy in chronic active hepatitis B, with significant reduction in serum ALT levels, increased OAS activity, and frequent clearance of HBeAg and HBV DNA (80). These effects were observed without notable adverse events, indicating that poly(A:U) is an effective and well-tolerated antiviral therapy.

Its adjuvant potential has been demonstrated by markedly enhanced antibody responses to recombinant hepatitis B surface antigen in mice, even at subimmunogenic antigen doses (81), and by improved protective immunity when combined with an inactivated rabies vaccine (82).

## 5 Poly(I:C) derivatives and clinical optimization

Poly(I:C) and its clinically optimized derivatives act as potent agonists of TLR3, promoting DC maturation and Th1-biased immune response, thereby supporting their development as immunotherapeutic adjuvants. However, early preclinical studies demonstrated that unmodified poly(I:C) undergoes rapid degradation by serum nucleases in primates, resulting in attenuated type I IFN production and reduced anti-tumor efficacy (83). These limitations, together with dose-dependent systemic toxicity, prompted the development of stabilized poly(I:C)-based formulations designed to improve pharmacological stability, safety, and immunostimulatory efficacy (84).

Poly(ICLC) is a synthetic dsRNA formulation composed of poly(I:C) complexed with poly-L-lysine and carboxymethylcellulose. This modification markedly increases resistance to serum nuclease-mediated hydrolysis and improves thermal stability relative to poly(I:C) alone, resulting in enhanced and sustained IFN induction in primates (11). Consequently, poly(ICLC) emerged as a more clinically applicable candidate for vaccine adjuvant and antiviral applications.

Further studies demonstrated that increasing the molecular size of both the polynucleotide and poly-L-lysine components of poly(ICLC) complexes enhanced resistance to ribonuclease-mediated degradation, which correlated with higher serum IFN levels in mice and rhesus monkeys. However, these improvements were accompanied by increased toxicity in murine models, reflected by lower LD_50_ values, underscoring an important stability–toxicity trade-off (85). In contrast, poly(I:C_12_U), another analogue, exhibits reduced toxicity but lower *in vivo* stability compared with poly(I:C) (1).

In nonhuman primates, poly(ICLC) enhanced HPV-specific cellular and humoral immune responses through robust Th1-biased immunity and the induction of CXCL9/CXCL10-driven innate chemokine responses, supporting effective antiviral immunity against HPV (1). Meanwhile, poly(I:C_12_U) (Ampligen) induces strong phenotypic and functional maturation of human myeloid DCs with reduced immunoregulatory IL-10 production, highlighting its potential utility in cancer-oriented immunotherapy (86).

Clinical evaluation of poly(ICLC) in a randomized, double-blinded, placebo-controlled trial demonstrated that the formulation is generally safe and well tolerated. Reported adverse events were predominantly mild to moderate, consisting mainly of Grade 1 injection-site reactions and transient systemic symptoms such as low-grade fever, chills, myalgias, fatigue, malaise, and headache; a single case of transient Grade 3 neutropenia was reported without clinical sequelae (87). Importantly, poly(ICLC) elicited robust yet transient innate immune activation, including IFN signaling, without compromising viral suppression in cART-treated individuals, supporting its use as an immunological adjuvant in therapeutic HIV vaccine strategies (87).

Despite substantial advances in synthetic dsRNA optimization, clinical translation remains incomplete and evidence supporting routine therapeutic implementation is still evolving. Among stabilized derivatives, poly(ICLC) (Hiltonol) has advanced most extensively into clinical evaluation, particularly as an immunomodulatory agent and vaccine adjuvant in oncology settings. Clinical studies indicate that poly(ICLC) can stimulate innate immune activation, enhance antigen presentation, and promote T-cell recruitment with an acceptable safety profile; however, most available evidence derives from early-phase trials with limited sample sizes, restricting conclusions regarding long-term efficacy and broad clinical applicability (7, 88). Consequently, poly(ICLC) has primarily been explored in combination with vaccines and immunotherapeutic approaches rather than as monotherapy.

Similarly, poly(I:C_12_U) (Ampligen) was developed to improve tolerability through selective modulation of dsRNA-mediated signaling pathways and has been investigated across infectious, oncologic, and immune-mediated conditions. Although clinical studies support favorable safety characteristics and reduced systemic toxicity relative to unmodified poly(I:C), therapeutic outcomes remain variable across disease indications. These findings emphasize that successful optimization of dsRNA agonists requires balancing immunostimulatory potency, pharmacokinetics properties, receptor selectivity, and systemic tolerability (89–91).

Collectively, current clinical experience suggests that future development of dsRNA-based adjuvants will depend on continued formulation refinement, biomarker-guided patient selection, optimized dosing schedules, and rational combination with vaccines or immunotherapies to maximize efficacy while minimizing inflammatory adverse effects.

## 6 Dosing and side effects of poly(I:C) and poly(A:U)

Dose optimization and safety considerations are critical for the translational application of synthetic dsRNA adjuvants. Understanding dosing parameters and associated side effects is therefore essential for agents such as poly(I:C) and poly(A:U). In preclinical studies, poly(I:C) administration in C57BL/6 mice produces dose-dependent adverse effects consistent with an acute inflammatory response. Doses of 2, 6, and 12 mg/kg induce reduced locomotor activity, weight loss, and significant alterations in body temperature, accompanied by elevated systemic and central expression of pro-inflammatory cytokines and markers of neuroinflammation (92). These findings highlight the narrow margin between immune activation and overt toxicity in preclinical models.

Beyond acute systemic effects, prenatal exposure to poly(I:C) leads to long-lasting neurobehavioral abnormalities in offspring, including schizophrenia-like phenotypes, gut microbiota dysbiosis, altered tryptophan metabolism, and neuroimmune dysregulation. These outcomes are associated with brain-specific epigenetic changes, including increased global histone deacetylase activity in offspring, suggesting that maternal immune activation induces enduring epigenetic reprogramming linked to later-life neuropathology (93, 94).

In humans, intravenous poly(I:C) is generally well tolerated at doses up to 6 mg/kg. Higher doses are associated with transient increases in liver enzymes and granulocyte counts, reflecting systemic immune activation. Fever exhibits a clear dose-dependent relationship with poly(I:C), while serum IFN levels remain largely unchanged across a tenfold dose range (1–10 mg/kg), suggesting differential regulation of downstream immune responses (95). Evidence from multiple studies supports the safety profile of stabilized formulations such as poly(ICLC) at therapeutically effective doses (96, 97). Reported adverse effects—including fever, fatigue, cytopenias, nausea, and hypotension—vary in incidence across studies and are generally manageable (96, 97). Hypotension and myalgia/arthralgia are dose-dependent and correlate with IFN induction, and higher doses can lead to severe musculoskeletal pain, marked hypotension, and rare but serious complications such as acute renal failure (98).

By contrast, poly(A:U) demonstrates a more favorable safety profile in humans. In a dose-escalation study of patients with advanced cancer, intravenous administration of poly(A:U) up to 450 mg was well tolerated, with no observed clinical, hematologic, or hepatic toxicity, while still inducing immunomodulatory effects such as enhanced NK cell activity (99). Repeated administration is also feasible; in a phase III trial of resected gastric cancer, weekly poly(A:U) doses over multiple cycles were well tolerated, supporting its safety for sustained clinical use (100).

Collectively, these findings underscore the context-dependent nature of TLR3 signaling, which can confer both protective antiviral effects and detrimental inflammatory or neurodevelopmental outcomes (101). This duality highlights the importance of careful dose selection and timing when employing TLR3 agonists such as poly(I:C) and poly(A:U) as antiviral therapeutics or vaccine adjuvants, including in cancer immunotherapy and infectious disease vaccines.

## 7 Discussion

The development of effective antiviral therapies and vaccines remains a critical research priority, particularly in the context of emerging infectious diseases. Synthetic dsRNA molecules such as poly(I:C) and poly(A:U) have been instrumental in elucidating dsRNA-sensing pathways and have directly informed the design of nucleic acid–based immunotherapies. Despite the emergence of newer RNA adjuvants and delivery platforms (102), dsRNA analogues remain valuable experimental and translational tools because of their well-defined mechanisms, reproducible immunostimulatory activity, and continued evaluation across preclinical and clinical settings. Despite these advances, rapid nuclease-mediated degradation of poly(I:C) remains a key limitation to its biological activity and continues to limit biological activity and remains an important target for formulation optimization (103). Consistent with its potent immunostimulatory profile, poly(I:C) induces strong IFN responses, whereas other dsRNAs, including poly(A:U), exhibit minimal activity (104, 105).

Liposome-encapsulated poly(I:C) accumulates in the liver of human liver-chimeric mice and exerts direct antiviral activity against persistent HBV infection (106). Stimulation of innate immune signaling by viral RNA mimetics activates IRF3-driven antiviral gene expression and contributes to suppression of HBV cccDNA, a key determinant of viral persistence (107). However, interpretation of these findings requires consideration of interspecies differences in antiviral immunity. Although humans and mice share broadly conserved antiviral defense pathways, hepatocyte responses differ substantially between species. Human hepatocytes produce high levels of IFN-λ and respond robustly to type III IFNs *in vivo*, whereas mouse hepatocytes rely predominantly on type I IFN signaling and exhibit limited responsiveness to IFN-λ (106, 108).

Poly(I:C) is widely used as a vaccine adjuvant due to its capacity to promote CD8 T-cell responses through TLR3 signaling; however, poly(I:C)-driven CD8 effector differentiation can occur independently of TLR3. Instead, this adjuvant activity is mediated through an IFN-α/β–dependent pathway, revealing additional mechanisms by which poly(I:C) enhances cell-mediated immunity beyond direct TLR3 engagement (109). Beyond vaccine applications, poly(I:C) is extensively employed to model viral infection and stimulate antitumor immunity. In contrast, poly(A:U), although generally less immunostimulatory, has attracted renewed interest because of its selective receptor engagement and potentially improved tolerability, supporting its use as a comparator in the rational development of dsRNA-based adjuvants (3, 110).

Although dsRNA analogues primarily signal through TLR3, the variable expression of TLR3 in tumor cells and the context-dependent functions of innate immune receptors in cancer underscore the importance of carefully evaluating dsRNA-based adjuvants in tumor vaccine strategies (3). Notably, poly(A:U)-induced IFN-β, mainly produced by CD11c^+^ cells through TLR3, plays a pivotal role in stimulating antitumor immune responses and controlling tumor growth in murine models (111). Further emphasizing its therapeutic potential, sustained local delivery of poly(I:C) using biodegradable hydrogels significantly reduced post-surgical tumor recurrence in multiple mouse models by transiently inducing IFN-α, modulating the tumor–wound microenvironment, promoting inflammatory monocyte recruitment, decreasing regulatory T cells, and enhancing tumor sensitivity to immune checkpoint blockade (112). Collectively, these observations indicate, preclinical and clinical studies demonstrate that poly(I:C) and its stabilized derivative poly(ICLC) function as potent danger-signal adjuvants that enhance antitumor immunity and improve tumor control (113).

In vaccine applications, poly(I:C) has demonstrated broad efficacy across infectious disease models, although formulation strongly influences both immunogenicity and tolerability. Nanoparticle-, emulsion-, and particulate-based delivery systems mitigating toxicity while preserving or enhancing immunogenicity. For example, poly(I:C) complexed with poly-L-lysine and encapsulated in lipid nanoparticles (LNPs) elicited robust humoral and T-cell responses in SARS-CoV-2 RBD–based vaccines (114), while calcium phosphate-functionalized poly(I:C) enabled dose-sparing and maintained protection against influenza virus infection (115). Synergistic innate immune activation has also been observed when poly(I:C) is co-delivered with antigen, particularly in particulate formulations containing inactivated virus (116). Moreover, combining poly(I:C) with the squalene-based nanoemulsion AddaVax enhanced influenza vaccine efficacy by promoting antigen uptake, DC activation, and lymph node trafficking (117).

However, despite encouraging preclinical outcomes, several challenges continue to limit the clinical translation of dsRNA agonists. Species-specific differences in innate immune sensing, variation in TLR3 and RIG-I-like receptor expression, differential cytokine responsiveness, and distinct pharmacokinetic profiles may substantially influence both the magnitude and quality of immune activation between animal models and humans (118, 119). Furthermore, preclinical studies employ controlled exposure conditions, genetically homogeneous populations, and surrogate immunological endpoints that may not accurately predict clinical performance. Translation is further complicated by the heterogeneity of existing clinical evidence, as studies of poly(I:C)-derived compounds are often exploratory, involve diverse administration routes and dosing regimens, and include relatively small patient cohorts, complicating cross-study comparisons and the establishment of standardized therapeutic protocols (3, 98, 120, 121). Moreover, immune stimulation sufficient to improve vaccine efficacy may partially overlap with pathways associated with systemic reactogenicity and inflammatory toxicity. Together, these challenges emphasize the importance of standardized clinical endpoints, improved delivery platforms, and systematic comparative evaluation across formulations to enable broader clinical implementation of dsRNA-based antiviral and vaccine strategies.

Nevertheless, advances in antigen targeting and formulation design continue to demonstrate the translational potential of synthetic dsRNA adjuvants. For example, targeting HIV epitopes to DEC205^+^ DCs revealed that poly(I:C) induced stronger costimulatory signaling and more durable, polyfunctional Th1-biased T-cell responses compared with other dsRNA adjuvants (122). These findings highlight how rational selection of RNA agonist and delivery strategies can maximize immunogenicity while limiting unnecessary inflammatory activation. Collectively, the accumulated evidence supports synthetic dsRNA agonists, particularly poly(I:C) and optimized derivatives, as versatile immunomodulatory platforms with continuing relevance for antiviral vaccination, cancer immunotherapy, and next-generation vaccine development.

## 8 Future directions

Future work should prioritize the rational engineering of dsRNA adjuvants that enable precise control over innate immune activation. While poly(I:C) and poly(A:U) have defined key principles of dsRNA sensing, important gaps remain in linking dsRNA structure to receptor selectivity, signaling magnitude, and cell-type–specific outcomes. Integrating structure–function analyses with advanced delivery systems and chemical modifications will be essential to optimize efficacy while minimizing inflammatory toxicity, particularly in clinical settings requiring repeated dosing.

Beyond single-agent formulations, combination adjuvant approaches and context-specific deployment warrant further investigation. Strategic pairing of dsRNA agonists with complementary innate or adaptive immune modulators may enhance response durability and enable dose sparing. In parallel, expanding dsRNA-based adjuvants to mucosal and personalized vaccine platforms will require a better understanding of tissue-specific innate sensing and tumor-intrinsic dsRNA signaling. Addressing these unresolved questions will be critical for translating dsRNA agonists into safe, adaptable, next-generation antiviral and anticancer vaccines.

## 9 Conclusion

Synthetic dsRNA analogues remain foundational tools for understanding antiviral innate immunity and for the development of vaccine adjuvants. Poly(I:C) and poly(A:U) exemplify how dsRNA structure influences receptor engagement, downstream signaling, and immune outcomes. Poly(I:C) exhibits broad and potent immunostimulatory activity, driving type I IFN responses and robust T-cell immunity across infectious disease and cancer models, while poly(A:U) provides a complementary profile with distinct signaling features and potential safety advantages.

Advances in formulation and targeted delivery have expanded the translational potential of dsRNA-based adjuvants by improving efficacy and mitigating toxicity. Comparative insights gained from these well-characterized molecules continue to inform the rational design of next-generation RNA immunomodulators. Collectively, poly(I:C) and poly(A:U) remain valuable benchmarks for dissecting dsRNA-mediated immunity and for guiding the development of antiviral and anticancer vaccine strategies.
